# Factors affecting contraceptive use among women seeking induced abortion in Hubei, China: a multicenter cross-sectional study

**DOI:** 10.3389/fpubh.2025.1650045

**Published:** 2026-01-05

**Authors:** Yun Tao, Chun Feng, Youling Zeng, Yujuan Li, Xin Du, Ling Zhang, Dan Li, Wenhua Zhang, Jiawei Kang, Juan Liu, Yuan Lu, Xia Sun, Yuanzhen Zhang

**Affiliations:** 1 Department of Obstetrics, Zhongnan Hospital of Wuhan University, Wuhan, Hubei, China; 2 Hubei Clinical Research Center for Prenatal Diagnosis and Birth Health, Wuhan, Hubei, China; 3 Wuhan Clinical Research Center for Reproductive Science and Birth Health, Wuhan, Hubei, China; 4 Department of Gynecology, Maternal and Child Health Hospital of Hubei Province, Wuhan, Hubei, China; 5 Department of Gynecology, Wuhan Children’s Hospital (Wuhan Maternal and Child Healthcare Hospital), Tongji Medical College Huazhong University of Science and Technology, Wuhan, Hubei, China; 6 Huanggang Maternal and Child Healthcare Hospital, Huanggang, Hubei, China; 7 Wuchang Maternal and Child Healthcare Hospital, Wuhan, Hubei, China; 8 Department of Obstetrics, Huangmei People’s Hospital, Huanggang, Hubei, China

**Keywords:** contraception, induced abortion, facilitators and barriers, women, China

## Abstract

**Background:**

Contraception plays a vital role in improving maternal and neonatal health by preventing unintended pregnancies. This study aimed to assess the history of contraceptive use and identify factors influencing contraceptive behavior among women seeking induced abortion in central China.

**Methods:**

A multicenter cross-sectional study was conducted in 25 hospitals across Hubei province. Data were collected using a structured questionnaire, and multivariable logistic regression was applied to evaluate factors associated with frequent contraceptive use.

**Results:**

Among 2,099 participants, only 53.1% reported frequent contraceptive use in the year prior to the current pregnancy. Frequent use was positively associated with higher education (postgraduate or above: OR = 4.81, 95% CI: 1.85–12.48), higher household income (>10,000 yuan/month: OR = 1.97, 95% CI: 1.38–2.82), longer interval since last abortion (≥12 months: OR = 2.07, 95% CI: 1.33–3.23), no history of abortion (OR = 2.35, 95% CI: 1.37–4.01), positive attitudes toward contraception (OR = 1.61, 95% CI: 1.32–1.96), and male-dominant decision-making in the household (OR = 1.45, 95% CI: 1.08–1.94). Only 2.3% of participants reported using long-acting reversible contraception. The findings align with the Theory of Planned Behavior, highlighting the roles of individual attitudes, subjective norms, and perceived behavioral control in shaping contraceptive practices.

**Conclusion:**

Contraceptive use among women seeking induced abortion remains suboptimal. Interventions should target improving attitudes toward contraception, engaging male partners, addressing socioeconomic barriers, and expanding access to diverse contraceptive options, especially for women with recent abortion history. These measures may help reduce unintended pregnancies and repeat abortions.

## 1 Introduction

Tens of millions of unintended pregnancies occur worldwide each year, the majority of which end in induced abortion. This phenomenon not only places substantial physical and psychological burdens on women but also consumes considerable healthcare and social resources. Providing effective immediate or post-abortion contraception (PAC) services to women seeking abortion is a key intervention to break the “unintended pregnancy–abortion” cycle and reduce the rate of repeat abortions. Despite the widespread availability of free contraceptives and PAC services in China the country continues to face a high rate of unintended pregnancies and subsequent abortions. Multiple studies have shown that even in regions with adequate access to contraceptive services, a large proportion of women seeking abortion did not use contraception―or did not use it correctly―during the sexual encounters that resulted in pregnancy (1, 2).

Contraception plays a vital role in improving maternal and neonatal health by preventing unintended pregnancies, which are associated with increased risks of adverse outcomes (3, 4). It is one of the most fundamental measure for safeguarding women’s reproductive health and fertility and is central to national health strategies and sustainable population development (5). In China, the *National Program for the Development of Chinese Women for the period 2021–2030* emphasizes improving reproductive health and reducing non-medical induced abortions (6). Recent research indicates that China’s contraceptive landscape now resembles that of many high-income countries (7). In 2019, the contraceptive prevalence rate (79.2%) and modern contraceptive prevalence rate (78.4%) exceeded global averages (51.9% and 47.7%) and even surpassed rates observed in some high-income settings (7).

Nonetheless, induced abortion remains a significant public health concern worldwide (7). In China, induced abortion is often perceived as a simple and safe gynecological procedure, yet its potential risks are frequently underestimated (8, 9). Approximately 10%–15% of women undergoing induced abortion experience complications, some of which may result in long-term infertility (10). National data indicate that the annual number of induced abortions has remained around 9.5 million over the past 5 years (11). During the same period, infertility among Chinese women of reproductive age has risen from 3.5% in 1997 to 16.4% in 2019 (12). With the relaxation of the two- and three-child policies, some individuals of reproductive age perceive contraception as less necessary. Additionally, contraceptive use in China is shaped by widespread misconceptions and stigma, especially concerning hormonal methods (13). In contrast, Netherlands―known for its progressive sexual health practices―reports low abortion rates (<5 per 1,000) and minimal teenage pregnancy, partly due in part to the widespread use of oral contraceptive (14).

Although previous studies in China have examined contraceptive behaviors among women seeking abortion, important determinants remain insufficiently explored. International evidence suggests that good contraceptive knowledge, positive attitudes, and effective partner communication are associated with higher contraceptive uptake (1). Among U.S. adolescents, risky behaviors such as binge drinking, cannabis use, casual sex, and certain demographics characteristics are linked to non-use of contraception (15, 16). Tu et al. (17) reported that nearly half of Chinese women seeking abortion became pregnant due to non-use of contraception, while most others relied on less or least effective methods both before and after abortion. Another study on long-acting reversible contraceptives (LARCs) found that intention to use these methods was influenced by parity, sexual activity, and marital status, with major barriers including concerns about future fertility, perceived health risks, irregular bleeding, and limited awareness (18).

While contraceptive behavior among women seeking induced abortion is a critical public health issue in China, key determinants—such as personal attitudes toward contraception, male partner involvement, and socioeconomic factors—remain underexamined. Therefore, this study aims to address these gaps by assessing contraceptive use history and identifying factors associated with contraceptive behaviors, thereby providing new insights into this vulnerable population.

## 2 Methods

### 2.1 Study design and setting

This multicenter cross-sectional survey employed stratified cluster random sampling. Hospitals were selected from all cities and autonomous prefectures in Hubei Province, central China, to ensure regional representativeness. Between September 15 and October 15, 2022, a total of 25 hospitals participated in the study. Women who underwent abortion for obstetrical or medical indications were excluded. Ethical approval was obtained from Ethics Committee of Zhongnan Hospital of Wuhan University (2022077K).

### 2.2 Study procedures

Data were collected using a structured questionnaire administered by healthcare providers. The questionnaire captured socio-demographic characteristics, reproductive and sexual history, contraceptive knowledge and attitudes, and contraceptive practices. Participants completed the survey using a secure online platform (Questionnaire Star) with assistance from trained nursing staff during waiting periods.

To ensure data quality, data collectors and supervisors underwent pre-testing and training covering topics such as confidentiality, participants’ rights, informed consent, study objectives, interview techniques, and standardized completion of questionnaires. Questionnaires were excluded if: I. Inconsistent answers; II. It takes less than 1 min or more than 1 h for questionnaire filling. All responses were anonymous and participation was voluntary.

### 2.3 Definitions

Contraception use: Frequency of contraception over the past year was assessed using a five-point Likert scale. Low-frequency use included “never,” “rarely,” and “occasionally”; high-frequency use included “regularly” and “every time.”

Contraceptive knowledge: Assessed using five multiple-choice questions (0 = incorrect, 1 = correct). Participants answering ≥75% correctly (mean score) were considered to have good knowledge.

Attitudes toward contraception: Assessed with a five-point Likert scale asking participants’ willingness to use effective contraception. Positive attitudes included responses “agree” or “strongly agree”; negative attitudes included “neutral,” “disagree,” or “strongly disagree.”

### 2.4 Data analysis

We conducted a study to examine the relationship between various characteristics and contraceptive use. To analyze the data, we used Chi-square analysis to compare the constituent ratios between the low-frequency use group and high-frequency use group. We included several candidate variables in our analysis, such as age, education, family income, marital status, number of abortions, age of first sexual intercourse, sex regularity, abortion interval, sexual determination, knowledge, and attitude. We only considered variables that were significant at the *p* < 0.2 level in the univariable analysis in the multivariable models. Additionally, we utilized multivariable logistic regression models to determine statistical significance if *p* < 0.05. All statistical analyses were conducted using Stata software.

## 3 Results

### 3.1 Characteristics of participants

Out of the total 2,322 participants in the survey, we obtained 2,099 valid questionnaires, resulting in an effective rate of 90.4%. In our study, we found that most characteristics differed in constituent ratios between the low-frequency use group and high-frequency use group. The mean age of the participants was 31.58 years (SD ± 5.4), with the predominant age group being 30–39 years (60.3%). Only 0.5% of participants in the high-frequency use group were under the age of 19, while 14.2% of participants in the low-frequency use group were under the age of 19. The majority of participants were married (84.8%) and had completed college education (98.3%). However, the composition ratio was different in the two groups. The majority of participants belonged to the family income group of 3,000–5,000 yuan per month, and this ratio also differed between the two groups. 9.0% of participants reported having had three or more previous abortions. Most participants were sexually irregular, with 5.3% reporting having had an induced abortion less than 6 months before their current pregnancy. Participants usually decided on contraceptive use through joint discussion during sexual activity (60.7%). Among the participants, 53.1% reported using contraceptives frequently within the last year before their current pregnancy (Table 1).

**Table 1:** Characteristics between low-frequency use and high frequency use of contraceptive.

Characteristics	Low-frequency use (%) (*n* = 984)	High-frequency use (%) (*n* = 1,115)	*p*
Age (years)			
<19	14 (14.2%)	6 (0.5%)	**0.009**
19–29	336 (34.1%)	330 (29.6%)	
30–39	561 (57.0%)	705 (63.2%)	
>39	73 (7.4%)	74 (6.6%)	
Education			
Primary school	58 (5.9%)	31 (2.7%)	**<0.001**
Junior high	310 (31.5%)	258 (23.1%)	
Senior high	254 (25.8%)	290 (26.0%)	
College	354 (36.0%)	507 (45.4%)	
Postgraduate or above	8 (0.8%)	29 (2.6%)	
Family income (Yuan per months)			
<3,000	220 (22.4%)	157 (14.1%)	**<0.001**
3,000–5,000	423 (43.0%)	444 (39.8%)	
5,001–10,000	252 (25.6%)	340 (30.5%)	
>10,000	89 (9.0%)	174 (15.6%)	
Marital status			
Unmarried	130 (13.2%)	117 (10.5%)	**0.021**
Married	813 (82.6%)	968 (86.8%)	
Divorced	41 (4.2%)	30 (2.7%)	
Number of abortions			
1	381 (38.7%)	432 (38.7%)	**0.003**
2	301 (30.6%)	399 (35.8%)	
3	200 (20.3%)	185 (16.6%)	
≥3	102 (10.4%)	87 (7.8%)	
First sexual intercourse age			
<15	12 (1.2%)	4 (0.3%)	**0.011**
16–20	319 (32.4%)	306 (27.4%)	
21–25	541 (55.0%)	682 (61.2%)	
26–30	102 (10.4%)	110 (9.9%)	
≥31	10 (1.0%)	13 (1.2%)	
Sex regularity			
Yes	166 (16.9%)	171 (15.3%)	0.34
No	818 (83.1%)	944 (84.7%)	
Abortion interval			
≤6 months	77 (7.8%)	35 (3.1%)	**<0.001**
6–12 months	94 (9.6%)	58 (5.2%)	
≥12 months	471 (47.9%)	610 (54.7%)	
No abortion history	342 (34.8%)	412 (37.0%)	
Who decides on contraception use?			
Female	225 (22.9%)	207 (18.6%)	**0.036**
Male	171 (17.4%)	221 (19.8%)	
Mutual decision	588 (59.8%)	687 (61.6%)	
Knowledge			
Poor	554 (56.3%)	660 (59.2%)	0.18
Good	430 (43.7%)	455 (40.8%)	
Attitude			
Negative	396 (40.2%)	308 (27.6%)	**<0.001**
Positive	588 (59.8%)	807 (72.4%)	

Bold values: *p*<0.05.

### 3.2 Knowledge and attitude of participants toward contraceptive use

Less than half of the participants had good knowledge about contraceptives (42.2%), and there were slightly more participants with poor knowledge in the high-frequency use group. Around half of the participants had a positive attitude toward contraceptives (66.5%), and there were more participants with positive attitudes in the high-frequency use group (Table 2).

**Table 2:** Factors of good contraceptive usage practice.

Characteristics	OR (95%CI)	*p*
Age (years)		
<19	Ref.	
19–29	2.20 (0.73–6.65)	0.16
30–39	2.81 (0.91–8.68)	0.072
>39	2.58 (0.80–8.36)	0.11
Education		
Primary school	Ref.	
Junior high	1.33 (0.82–2.16)	0.25
Senior high	1.75 (1.07–2.87)	**0.026**
College	1.90 (1.16–3.14)	**0.011**
Postgraduate or above	4.81 (1.85–12.48)	**0.001**
Family Income (Yuan per months)		
<3,000	Ref.	
3,000–5,000	1.30 (1.00–1.69)	**0.048**
5,001–10,000	1.61 (1.21–2.14)	**0.001**
>10,000	1.97 (1.38–2.82)	**<0.001**
Marital status		
Unmarried	Ref.	
Married	1.07 (0.68–1.68)	0.76
Divorced	0.71 (0.37–1.36)	0.30
Number of abortions		
1	Ref.	
2	1.43 (0.97–2.10)	0.07
3	1.03 (0.68–1.57)	0.88
≥3	0.99 (0.62–1.60)	0.98
First sexual intercourse age		
<15	Ref.	
16–20	1.99 (0.60–6.68)	0.26
21–25	2.36 (0.71–7.87)	0.16
26–30	1.71 (0.50–5.89)	0.39
≥31	2.80 (0.63–12.40)	0.17
Abortion interval		
≤6 months	Ref.	
6–12 months	0.99 (0.58–1.71)	0.98
≥12 months	2.07 (1.33–3.23)	**0.001**
No abortion history	2.35 (1.37–4.01)	**0.002**
Who decides on contraception use?		
Female	Ref.	
Male	1.45 (1.08–1.94)	**0.012**
Mutual decision	1.15 (0.91–1.45)	0.23
Knowledge		
Poor	Ref.	
Good	0.85 (0.71–1.02)	0.074
Attitude for contraceptives		
Negative	Ref.	
Positive	1.61 (1.32–1.96)	**<0.001**

Bold values: *p*<0.05.

### 3.3 The factors associated with contraceptive use

In a multivariable logistic regression analysis, several factors were found to be significantly associated with contraceptive use among women. These factors included attitude toward contraceptives, sexual determination, abortion interval, family income, and education.

Women who had a positive attitude toward contraceptives had 1.61 times higher odds (95% CI: 1.32, 1.96) of using contraceptives compared to those who did not. Interestingly, women whose partner usually decided whether to use contraceptives had 1.45 times higher odds (95% CI: 1.08, 1.94) of using contraceptives compared to women who made the decision by themselves. Women who had induced abortions more than or equal to 12 months ago or had no abortion history had 2.07 times (95% CI: 1.33, 3.23) and 2.35 times (95% CI: 1.37, 4.01) higher odds of contraceptive use, respectively, compared to those who had an induced abortion within the last 6 months. Women from higher-income families had 1.97 times (95% CI: 1.38, 2.82) higher odds of using contraceptives frequently compared to those from low-income households. Finally, higher educational levels were associated with higher odds of contraceptive use. Women with postgraduate or above degrees had 4.81 times (95% CI: 1.85, 12.48) higher odds of contraceptive use compared to those who only completed primary school (Table 2).

### 3.4 Contraceptive use

Among the participants who underwent induced abortion, 50.1% of them had not been using any form of contraception prior to the pregnancy. Among those who were not using contraceptives, 39.2% reported that it was an unplanned sexual encounter, 27.1% had a mentality of relying on luck, 13% had planned to have children but had a change of plans. Additionally, 5.2% of the participants reported not knowing how to use contraceptives, and 3.9% reported that their partners had refused to use contraceptives (Table 3).

**Table 3:** Reasons for not using contraceptives among women seeking induced abortion.

Reasons	Percentage
Unplanned sex	39.21%
The mentality of relying on luck	27.12%
Had planned to have children	13.00%
Fear of side effect	9.42%
Not knowing how to use contraceptive	5.22%
Partner refusal	3.89%
Other factors	1.83%
Forced sex	0.31%

Among the participants, 49.9% used contraceptives in this pregnancy, indicating a failure rate. Among those who experienced contraceptive failure, 30.0% used condoms, 25.2% used calendar methods such as rhythm or standard days, and 23.3% used withdrawal before ejaculation. Emergency contraceptives were used by 14.0% of those who experienced contraceptive failure. In addition, 2.3% of them used intrauterine devices and 1.8% used oral contraceptive pills. None of the participants who experienced contraceptive failure had used implants or undergone sterilization (Table 4).

**Table 4:** Types of contraceptives used among women seeking induced abortion.

Types	Percentage
Condom	29.96%
Calendar or rhythm method	25.19%
Withdrawal	23.28%
Emergency contraception	14.03%
Contraceptive for external use	3.44%
Intra Uterine device (IUD)	2.29%
Oral contraceptive pills	1.81%
Subdermal implants	0%
Sterilization	0%

## 4 Discussion

This is a large-scale, multi-center study examined factors associated with contraceptive use among women seeking induced abortion in central China. Among the 2,099 participants, only 53.1% reported frequent contraceptive use during the year prior to the current pregnancy. This rate aligns with the findings from the US, where 40% of adolesecents report “always” using contraception and 34% report “sometimes” using it (19). In Iran, 63.5% abortion-seeker women were using contractive method, which is higher than our finding (20). In comparison, 63.5% of abortion-seeking women in Iran reported contraceptive use, which is higher than our findings, whereas the rate in Ethiopia was lower at 41.3% (1). These variations highlight the influence of regional socio-demographic factors on contraceptive prevalence (7). In China, the prevalence of modern contraception has reached 78.4%, exceeding the global average and even surpassing some middle- and high-income countries (7).

Education level emerged as a significant predictor of frequent contraceptive use, consistent with previous studies, as lower educational attainment may limit knowledge about contraception and abortion-related risks (21–23). Interestingly, women with good contraceptive knowledge were not necessarily more likely to use contraception frequently, differing from prior findings (1, 24). Household income was also positively associated with contraceptive use, indicating that greater resources enhance women’s ability to access and utilize contraception effectively.

Attitudes toward contraception played a central role. Women with positive attitudes were more likely to use contraception consistently, supporting the importance of reducing stigma and improving communication with partners and healthcare providers (1, 24). Individuals who hold positive attitudes toward contraception are more likely to use it consistently and effectively. Positive attitudes toward contraception can reduced stigma surrounding contraceptive use, and improved communication about contraception with partners and healthcare providers.

This study highlights the importance of considering a woman’s abortion history when promoting contraceptive use. In our survey, women who have never had an abortion or who had an abortion more than or equal to 12 months ago were more likely to use contraceptives frequently. The incidence of induced abortion may be influenced by the level of emphasis placed on contraception and the effectiveness of its use. Women who have had a long interval between induced abortions or no history of induced abortion may perceive induced abortion as an incidental event. In China, post-abortion care programs are in place to provide women with access to contraceptives and assistance in their use (25). Previous research suggests that women who have had an abortion may have a greater awareness of the importance of preventing unintended pregnancy and may be more likely to use contraceptives in the future (26).

In this study, individual barriers to using contraceptives were found to be important factors. Unplanned sex, relying on luck, and changes in plans to have children were among the main individual barriers identified. Unplanned sex can make it difficult for individuals to have access to and use contraceptives effectively. Relying on luck may also reflect a lack of understanding about the risks of unintended pregnancy and the importance of using contraceptives consistently. Changes in plans to have children, such as a desire to become pregnant, can also impact contraceptive use. Other individual barriers that have been identified in previous research include fear of side effects, lack of access to contraceptives, and cultural or religious beliefs (23).

In terms of contraceptive options, this study found that many women who sought induced abortions reported failure of the contraceptive method they had chosen. The top three contraceptive methods that were associated with contraceptive failure among this population were condoms, calendar or rhythm method, and withdrawal. It is important to note that these findings do not necessarily mean that these methods are ineffective for all individuals, but rather that they may not be the best options for some women. Additionally, it highlights the need for increased education and access to a wider range of effective contraceptive options.

In this study, the Theory of Planned Behavior (TPB) was used to interpret the determinants of contraceptive behavior among women seeking induced abortion. According to TPB, behavioral intention—and ultimately behavior—is shaped by three key components: attitudes, subjective norms, and perceived behavioral control (27–29). Our empirical findings align closely with these theoretical pathways. First, attitudes toward contraception played a central role. Women with more positive attitudes were significantly more likely to report frequent contraceptive use (OR = 1.61), consistent with TPB’s assertion that favorable attitudes strengthen behavioral intention (27). Second, subjective norms were reflected in partner involvement. Women from male-dominant decision-making households had higher odds of using contraception, indicating that partner approval and relationship dynamics shape contraceptive choices. This supports TPB’s emphasis on social expectations and interpersonal influences (30, 31). Third, perceived behavioral control was mirrored in socioeconomic indicators. Higher education and family income were positively associated with contraceptive use, suggesting that greater resources and knowledge enhance women’s perceived ability to access and consistently use effective methods (28). Overall, the study’s findings strongly support the TPB framework, showing that contraceptive behavior in this population is jointly influenced by individual attitudes, interpersonal norms, and perceived capability. These insights highlight the need for interventions that not only improve knowledge and attitudes but also engage male partners and address structural barriers that limit women’s autonomy in contraceptive decision-making.

This study has several limitations that should be taken into consideration when interpreting the results. One of the limitations is the potential for the participants are women who are seeking for induced abortion, extrapolation of the results to women of reproductive age requires more consideration. Additionally, due to the cross-sectional design of the study, it was not possible to establish a cause-and-effect relationship between the independent and outcome variables. Future research utilizing longitudinal study designs may provide more insight into the relationships between these variables.

## 5 Conclusion

This study aimed to assess the history of contraceptive use and identify the factors influencing contraceptive behavior among women seeking induced abortion in China. The empirical findings effectively support this objective by demonstrating that only half of the participants practiced frequent contraceptive use and by identifying several determinants that significantly shape contraceptive behaviors. Higher educational attainment, greater household income, a longer interval since the last abortion, positive attitudes toward contraception, and partner involvement were all shown to facilitate contraceptive use, whereas women with a recent history of abortion were less likely to adopt effective methods. These findings contribute to addressing the previously identified gap by clarifying the socio-demographic, attitudinal, and interpersonal factors that influence contraceptive behavior within this population. They also highlight the complexity of contraceptive decision-making and underscore the need for targeted strategies. Interventions that strengthen contraceptive knowledge among low-income women, improve attitudes toward modern methods, and engage male partners may help increase contraceptive uptake. Tailored counseling and improved access to a broader range of contraceptive options are particularly important for women who have recently undergone abortion. Overall, the study provides evidence-based insights that can inform more effective, personalized contraceptive services, ultimately helping to reduce unintended pregnancies and the burden of repeat induced abortions.

## Data Availability

The data are not publicly available due to privacy or ethical restrictions. However, data may be available from the corresponding author upon reasonable request.

## References

[1] Alemu L Ambelie YA Azage M. Contraceptive use and associated factors among women seeking induced abortion in Debre Marko’s town, Northwest Ethiopia: a cross-sectional study. Reprod Health. (2020) 17:97. doi: 10.1186/s12978-020-00945-4, 32552736 PMC7301557

[2] Lamina MA. Prevalence of abortion and contraceptive practice among women seeking repeat induced abortion in Western Nigeria. J Pregnancy. (2015) 2015:486203. doi: 10.1155/2015/486203, 26078881 PMC4452495

[3] GBD 2019 Diseases and Injuries Collaborators. Global burden of 369 diseases and injuries in 204 countries and territories, 1990-2019: a systematic analysis for the global burden of disease study 2019. Lancet. (2020) 396:1204–22. doi: 10.1016/S0140-6736(20)30925-9 33069326 PMC7567026

[4] Conde-Agudelo A Rosas-Bermudez A Castaño F Norton MH. Effects of birth spacing on maternal, perinatal, infant, and child health: a systematic review of causal mechanisms. Stud Fam Plan. (2012) 43:93–114. doi: 10.1111/j.1728-4465.2012.00308.x, 23175949

[5] Finlay JE. Women’s reproductive health and economic activity: a narrative review. World Dev. (2021) 139:105313. doi: 10.1016/j.worlddev.2020.105313

[6] Nie J-B. Non-medical sex-selective abortion in China: ethical and public policy issues in the context of 40 million missing females. Br Med Bull. (2011) 98:7–20. doi: 10.1093/bmb/ldr015, 21596712

[7] Haakenstad A Angelino O Irvine CMS Bhutta ZA Bienhoff K Bintz C . Measuring contraceptive method mix, prevalence, and demand satisfied by age and marital status in 204 countries and territories, 1970-2019: a systematic analysis for the global burden of disease study 2019. Lancet. (2022) 400:295–327. doi: 10.1016/S0140-6736(22)00936-9, 35871816 PMC9304984

[8] Liu J Wu S Xu J Temmerman M Zhang WH INPAC Group. Is repeat abortion a public health problem among Chinese adolescents? A cross-sectional survey in 30 provinces. Int J Environ Res Public Health. (2019) 16:794. doi: 10.3390/ijerph16050794, 30841501 PMC6427833

[9] Yokoe R Rowe R Choudhury SS Rani A Zahir F Nair M. Unsafe abortion and abortion-related death among 1.8 million women in India. BMJ Glob Health. (2019) 4:e001491. doi: 10.1136/bmjgh-2019-001491, 31139465 PMC6509605

[10] Qureshi Z Mehrtash H Kouanda S Griffin S Filippi V Govule P . Understanding abortion-related complications in health facilities: results from WHO multicountry survey on abortion (MCS-A) across 11 sub-Saharan African countries. BMJ Glob Health. (2021) 6:e003702. doi: 10.1136/bmjgh-2020-003702, 33514590 PMC7845704

[11] Jin Y Hu W. Diverging destinies: changing trends of induced abortion in China. China Popul Dev Stud. (2023) 7:1–32. doi: 10.1007/s42379-023-00129-037193368

[12] Burger M Einspieler C Niehaus DJH Unger M Jordaan ER. Maternal mental health and infant neurodevelopment at 6 months in a low-income south African cohort. Infant Ment Health J. (2022) 43:849–63. doi: 10.1002/imhj.22021, 36268625 PMC9828192

[13] Lai RJC. Every medicine is somewhat poisonous: understanding the reluctance to use oral contraceptives among unmarried women seeking abortion in China. Contraception. (2023) 119:109917. doi: 10.1016/j.contraception.2022.11.008, 36473512

[14] Apter DJ. International perspectives: IUDs and adolescents. J Pediatr Adolesc Gynecol. (2019) 32:S36–42. doi: 10.1016/j.jpag.2019.04.009, 31585617

[15] Chernick LS Siden JY Bell DL Dayan PS. A qualitative assessment to understand the barriers and enablers affecting contraceptive use among adolescent male emergency department patients. Am J Mens Health. (2019) 13:1557988319825919. doi: 10.1177/1557988319825919, 30819063 PMC6440070

[16] Chernick LS Chun TH Richards R Bromberg JR Ahmad FA McAninch B . Sex without contraceptives in a multicenter study of adolescent emergency department patients. Acad Emerg Med. (2020) 27:283–90. doi: 10.1111/acem.13867, 31596987 PMC7141959

[17] Tu P Hu D Wu S Li J Jiang X Pei K . Characteristics and contraceptive practices among Chinese women seeking abortion: a multicentre, descriptive study from 2019 to 2021. BMJ Sex Reprod Health. (2024) 50:252–61. doi: 10.1136/bmjsrh-2023-202181, 38702180 PMC11503149

[18] Luo Z Gao L Anguzu R Zhao J. Long-acting reversible contraceptive use in the post-abortion period among women seeking abortion in mainland China: intentions and barriers. Reprod Health. (2018) 15:85. doi: 10.1186/s12978-018-0543-2, 29793501 PMC5968602

[19] Bexhell H Guthrie K Cleland K Trussell J. Unplanned pregnancy and contraceptive use in Hull and East Yorkshire. Contraception. (2016) 93:233–5. doi: 10.1016/j.contraception.2015.10.004, 26519645 PMC4766045

[20] Bayrami R Javadnoori M. Comparison of the contraceptive use and its related factors among women seeking repeat and first-time induced abortions in Iran. Nurs Midwifery Stud. (2015) 4:e17529. doi: 10.17795/nmsjournal17529, 25830153 PMC4377525

[21] Liang M Simelane S Fortuny Fillo G Chalasani S Weny K Salazar Canelos P . The state of adolescent sexual and reproductive health. J Adolesc Health. (2019) 65:S3–s15. doi: 10.1016/j.jadohealth.2019.09.015, 31761002

[22] Sarder A Islam SMS Maniruzzaman Talukder A Ahammed B. Prevalence of unintended pregnancy and its associated factors: evidence from six south Asian countries. PLoS One. (2021) 16:e0245923. doi: 10.1371/journal.pone.0245923 33524018 PMC7850499

[23] Maravilla JC Betts KS Couto ECC Alati R. Factors influencing repeated teenage pregnancy: a review and meta-analysis. Am J Obstet Gynecol. (2017) 217:527–545.e531. doi: 10.1016/j.ajog.2017.04.021 28433733

[24] Liu R Dong X Ji X Chen S Yuan Q Tao Y . Associations between sexual and reproductive health knowledge, attitude and practice of partners and the occurrence of unintended pregnancy. Front Public Health. (2022) 10:1042879. doi: 10.3389/fpubh.2022.1042879 36684880 PMC9846217

[25] Wang H Liu Y Xiong R. Factors associated with seeking post-abortion care among women in Guangzhou, China. BMC Womens Health. (2020) 20:120. doi: 10.1186/s12905-020-00980-0, 32522197 PMC7288524

[26] Ameyaw EK Budu E Sambah F Baatiema L Appiah F Seidu AA . Prevalence and determinants of unintended pregnancy in sub-Saharan Africa: a multi-country analysis of demographic and health surveys. PLoS One. (2019) 14:e0220970. doi: 10.1371/journal.pone.0220970, 31398240 PMC6688809

[27] Ajzen I. The theory of planned behaviour: reactions and reflections. Psychol Health. (2011) 26:1113–27. doi: 10.1080/08870446.2011.613995, 21929476

[28] Ajzen I. Perceived behavioral control, self-efficacy, locus of control, and the theory of planned behavior. J Appl Soc Psychol. (2002) 32:665. doi: 10.1111/j.1559-1816.2002.tb00236.x

[29] Fishbein M Ajzen I. Predicting and changing behavior: the reasoned action approach. New York, NY: Psychology Press (2011).

[30] Courneya KS Plotnikoff RC Hotz SB Birkett NJ. Social support and the theory of planned behavior in the exercise domain. Am J Health Behav. (2000) 24:300–8. doi: 10.5993/AJHB.24.4.6

[31] Kariman N Hashemi SSB Ghanbari S Pourhoseingholi MA Alimoradi Z Fakari FR. The effect of an educational intervention based on the theory of planned behavior on childbearing intentions in women: a quasi-experimental study. J Educ Health Promot. (2020) 9:96. doi: 10.4103/jehp.jehp_2_20, 32509904 PMC7271907

